# Nationwide population-based study reveals increased malignancy risk in taiwanese liver transplant recipients

**DOI:** 10.18632/oncotarget.11965

**Published:** 2016-09-10

**Authors:** Yung Fong Tsai, Hsiu Pin Chen, Fu Chao Liu, Shih Hao Liu, Chun Yu Chen, Chih Wen Cheng, Jr Rung Lin

**Affiliations:** ^1^ Department of Anesthesiology, Chang Gung Memorial Hospital, Taoyuan, Taiwan; ^2^ College of Medicine, Chang Gung University, Taoyuan, Taiwan; ^3^ Clinical Informatics and Medical Statistics Research Center and Graduate Institute of Clinical Medicine, Chang Gung University, Taoyuan, Taiwan

**Keywords:** de novo malignancy, immunosuppression, mortality, post-transplant malignancy, recurrent malignancy

## Abstract

Post-transplant malignancy is a major cause of late mortality for liver transplant recipients (LTRs). This nationwide population-based cohort study investigated the cancer type, incidence, and risk factors associated with post-transplant malignancies in 2938 Taiwanese LTRs who underwent transplantation between 1998 and 2012. Data from the National Health Insurance Research Database were extracted on the basis of the International Classification of Disease, Ninth Revision, Clinical Modification codes. Among these patients, 284 post-transplant malignancies were diagnosed. These included 99 *de novo* malignancies among 98 patients, yielding a standardized incidence ratio of 2.17 (95% CI, 1.76 to 2.64) compared to the general population. The most common malignancies were infection related liver cancer (19.39%), oropharyngeal cancer (19.39%), non-Hodgkin's lymphoma (9.18%), and esophageal cancer (5.10%), as well as non-infection-related prostate cancer (6.12%). Patients with recurrent malignancies had the highest mortality. Furthermore, 186 recurrent malignancies relapsed, and the commonly affected organs were the liver (83.33%), lung (4.84%), bone and bone marrow (4.30%), and intrahepatic bile ducts (2.69%). Old age, the male sex, liver cirrhosis, hepatitis B, peptic ulcer, diabetes mellitus, and pre-existing cancer were all risk factors associated with post-transplant malignancies. Recipients with biliary atresia or urea cycle metabolism disorders were protected from post-transplant malignancies. Our data revealed a significantly increased risk of malignancies in Taiwanese LTRs and suggest implementation of a careful malignancy-surveillance program and immunosuppression-minimizing strategy for high-risk patients.

## INTRODUCTION

Liver transplantation is an effective and promising choice to cure patients with end-stage liver diseases or early inoperable hepatocellular carcinoma (HCC) [1], who otherwise have less than one year to live [2]. Advances in surgical techniques and medical care have lately yielded a one-year survival rate after liver transplantation of more than 80%-90% [3, 4]. However, liver transplant recipients (LTRs) still have a lower probability of long-term survival than the age-matched general population [5]. Closely followed by common causes of death in the general population, hepatic-related, post-transplant malignancies account for 22% of LTR mortality [6]. Several epidemiological studies have revealed a 2- to 4-fold higher risk of post-transplant *de novo* malignancies in LTRs than in the general population [5, 7–9]. Immunosuppression is an essential contributor to oncogenesis in LTRs, particularly for those with infection-related cancers. This viewpoint was firmly supported by the similar spectrum of cancer risk seen in LTRs and patients with acquired immunodeficiency syndrome (AIDS) [10].

Malignancies occurring after solid organ transplantations, other than liver, are mainly *de novo* cancers because recipients with active malignancies are typically excluded. Recipients with HCC typically have poor survival and considerably elevated recurrence rates because of poor patient selection [13, 14]. The Milan criteria have been widely used for candidate selection since 1996, and five-year survival obtained using these criteria was more satisfactory than that obtained using others (87% *vs*. 62%) [15]. In Taiwan, the first liver transplantation for HCC was performed in 1999. Because transplantation for HCC has since become well accepted in Taiwan, recurrent malignancies among LTRs could become an increasingly serious problem.

Few studies have summarized and compared *de novo* and recurrent malignancies after liver transplantation. Of these, many were limited by a single-center design or small cohort population and they rarely analyzed risk factors associated with post-transplant malignancies in LTRs [9, 11, 12, 16]. Determining risk factors for post-transplant malignancies provides a basis for preventive and therapeutic interventions and could promote early diagnosis and improve long-term survival. The present study aimed to retrospectively determine the patterns of post-transplant malignancies and associated risk factors. The spectrum and prevalence of post-transplant malignancies vary among ethnicities and geographical regions. We herein analyzed the incidence of *de novo* and recurrent malignancies in Taiwanese LTRs over a 14.5-year observation period compared to the general population. As per our review of relevant data, our study is the first to provide insights into cancer etiology and support the development of evidence-based strategies for identifying high-risk recipients by examining patients of Chinese ethnicity.

## MATERIALS AND METHODS

### Data source

This population-based cohort study retrospectively analyzed data from Taiwan's National Health Insurance Research database (NHIRD). It enrolled more than 99.9% of the entire population of 23.32 million Taiwanese residents, and the Bureau of National Health Insurance (NHI) is affiliated to more than 93% medical institutions in Taiwan. The claims data retrieved from the NHIRD were computerized and de-identified. The NHIRD comprises enrollment information, inpatient and outpatient claims for reimbursement, and prescription drugs and procedures. Furthermore, the database provides disease diagnostic codes according to the International Classification of Disease, Ninth Revision, Clinical Modification (ICD-9-CM). In addition, the annual incidence rate of cancer in the general population was obtained from the Taiwan National Cancer Registry (http://www.hpa.gov.tw/BHPNet/Web /Stat/StatisticsShow.aspx?No = 200911300001, and http://www.hpa.gov.tw/BHPNet/ Web/ Stat /Statistics.aspx).

The study protocol conformed to the ethical guidelines of the 1975 Declaration of Helsinki as reflected in *a priori* approval by the Institutional Review Board of Chang Gung Memorial Hospital (Registration number: IRB 103-0102B) and NHIRD research committee (Registration number: NHIRD-103-103). Personal information and medical records were identified and anonymized; therefore, informed consent from the LTRs was unavailable.

### Definition and selection of the study population

The target LTRs were identified on the basis of the ICD-9-CM code 996.82 or V427 from the catastrophic illness database. In addition, we excluded patients who did not match the operation code of liver transplant surgery (505, 75020A, or 75020B) within this period. Finally, 2938 LTRs were included in this cohort study.

Preoperative comorbidities were identified using a preset definition of at least one recorded inpatient department diagnosis or five recorded outpatient department diagnoses before transplantation using the claimed codes. The specific ICD-9-CM codes for identification are as follows: liver cirrhosis (ICD-9-CM 571.2, 571.5, and 571.6), chronic hepatitis (ICD-9-CM 070, 571, and 573.3), alcoholic hepatitis (ICD-9-CM 571.2 and 571.3), hepatitis B (ICD-9-CM 070.2, 070.3, V0261, and V0269), hepatitis C (ICD-9-CM 070.41, 070.44, 070.51, 070.54, 070.7, and V262), hypertension (ICD-9-CM 401-405), diabetes mellitus (ICD-9-CM 250), peptic ulcer (ICD-9-CM 531-533), obesity (ICD-9-CM 278), ascites (ICD-9-CM 789.5), hepatic coma (ICD-9-CM 070.0, 070.2, 070.4, 070.6, 70.2, 70.41, 572.2, 070.71, and 348.3), renal failure (ICD-9-CM 584-586), pulmonary disease (ICD-9-CM 490-496), hypercholesterolemia (ICD-9-CM 272), malignancies (ICD-9-CM 140-208.91), biliary atresia (ICD-9-CM 751.61), Wilson disease (ICD-9-CM 275.1 and E8301), glycogen storage disease (ICD-9-CM 271.0), urea cycle metabolism disorders (ICD-9-CM 270.6), bacteremia (ICD-9-CM 038 and 998.5), and postoperative bleeding (ICD-9-CM 998.0-998.2). Death was defined as the matched death code or termination from the NHI program.

### Definition of primary outcome

The primary outcomes were post-transplant malignancies and long-term mortality in the LTRs. Post-transplant cancers were defined according to the recorded ICD-9-CM codes 140-208.91 in the same catastrophic illness database after liver transplantation. Benign neoplasms (ICD-9-CM 210-229) and *in situ* carcinoma (ICD-9-CM 230-234) were not included as post-transplant malignancies. To avoid bias in the post-transplant malignancy rate, which may be caused by an increased pre-transplantation examination, patients diagnosed with cancer within 3 months postoperatively were excluded. Furthermore, *de novo* cancer is defined as the first occurrence without a prior existence of that cancer in the body. Recurrent malignancy is defined as cancer reappearance after a remission interval when the cancer cannot be detected. The relapsed cancer can develop at the original location or in other parts of the body. Variables comprised demographic characteristics, and comorbidities were analyzed for the risk factors for post-transplant malignancies.

The survival time of the LTRs was defined as the number of days from transplantation to death. For comparison of long-term survival LTRs were categorized into four groups: 1. Malignancy-free group, no cancer diagnosed throughout the study period; 2. Malignancy-cured group, preoperative malignancies were not diagnosed postoperatively. 3. De novo group, the first occurrence of cancer postoperatively; and 4. Recurrent group, the postoperative development of relapsed cancer, which existed preoperatively.

### Statistical analysis

Data are expressed as means ± standard deviation, except the representatives of the survival time that are presented as mean ± standard error (SE). Statistical analysis was performed using SAS (version 9.3; SAS Institute Inc., Cary, NC, USA). The variables for comparing the risk factors between the malignancy and non-malignancy groups were expressed as competing risk cox regression model with control mortality. Patient death or loss to follow-up in the NHIRD was identified as patient mortality. A *p*-value of < 0.05 was considered significant, and all statistical analyses were two-sided.

## RESULTS

### Characteristics of the LTRs

We recruited 2938 Taiwanese LTRs who underwent transplantation between July 1998 and December 2012; patient characteristics are described in Table 1. Of all LTRs, 2068 were males, and 870 were females; 341 were pediatric patients less than 18 years old. The average age at the time of the transplant was 46.42 ± 17.68 years (range, 4.44 months to 75.15 years; median, 51.76 years). The average follow-up interval was 3.79 ± 3.26 years, with a total follow-up of 14176 person-years. The most common follow-up duration was between 1 and 5 years, and 6.19% of the LTRs were followed for more than 10 years. Furthermore, 1440 LTRs were diagnosed with preoperative malignancies, 1313 of these with liver cancer (91.18%). The other 1498 LTRs were cancer-free preoperatively. 2484 patients had liver cirrhosis. Among the 2761 cases of chronic hepatitis, 1416 were HBV related, 662 were HCV related, and 565 were alcohol related.

**Table 1:** Characteristics of Taiwan liver transplant recipients from 1998 through 2012

N=2938	N, Mean	%, SD
**Sex**		
Male	2068	70.39
Female	870	29.61
**Age (years)**^†^	46	17.68
**Age group**		
< 18	341	11.61
≧ 18	2597	88.39
**Follow-up time (years)**^†^	3.79	3.26
<1	623	21.20
1-5	1466	49.90
5-10	667	22.70
>10	182	6.19
**Person-years of follow-up**	14176	
**Pre-op cancer**		
Yes	1440	49.01
No	1498	50.99
**Pre-op HCC**		
Yes	1313	44.69
No	1625	55.31
**Liver cirrhosis**	2484	84.55
**Chronic hepatitis**		
Yes	2761	93.98
HBV-related	1416	48.20
HCV-related	662	22.53
Alcohol-related	565	19.23
No	177	6.02
**Biliary atresia**		
Yes	284	9.67
No	2654	90.33
**Wilson's disease**		
Yes	35	1.19
No	2903	98.81
**Glycogen storage disease**		
Yes	28	0.95
No	2910	99.05
**Urea cycle metabolism disorders**		
Yes	16	0.54
No	2922	99.46

† Values are mean and standard deviation. HCC, hepatocellular carcinoma.

### Risk of post-transplant malignancies in the LTRs compared with the general Taiwanese population

The annual incidences of malignancies for the estimated Taiwanese population of 23.32 million as of 2012 are listed in Table 2. The average annual incidence of *de novo* malignancies from 1998 to 2012 was approximately 0.32% in the general population. In the LTRs, the proportion of total post-transplant malignancies was 9.67%, the proportion of *de novo* malignancies was 3.34% (98/2938), and that of recurrent malignancies was 6.33% (186/2938). Moreover, the standardized incidence ratio (SIR) of *de novo* cancer between LTRs and the general population was 2.17 (95% CI, 1.76 to 2.64).

**Table 2:** Annual number of patients with de novo malignancy in cohort study and general Taiwan population from 1998 through 2012

Year	General population		Cohort population			
	*De novo* malignancy	Population number	*De novo*malignancy	Recurrent malignancy	Transplant recipient	Cumulative person-years of follow-up
1998	52207	21928591	0	0	12	180
1999	56323	22092387	0	0	29	586
2000	59116	22276672	0	0	40	1106
2001	61606	22405568	0	0	64	1874
2002	63736	22520776	0	3	70	2644
2003	62542	22604550	4	2	134	3984
2004	67895	22689122	9	8	108	4956
2005	68907	22770383	3	7	158	6220
2006	73293	22876527	8	7	179	7473
2007	75769	22958360	5	16	227	8835
2008	79818	23037031	8	12	289	10280
2009	87189	23119772	13	25	326	11584
2010	90649	23162123	16	41	406	12802
2011	92682	23224912	14	46	478	13758
2012	96694	23315822	18	19	418	14176
Total	1088425		98	186	2938	

### Incidence and mortality of post-transplant malignancies

**Figure 1:**
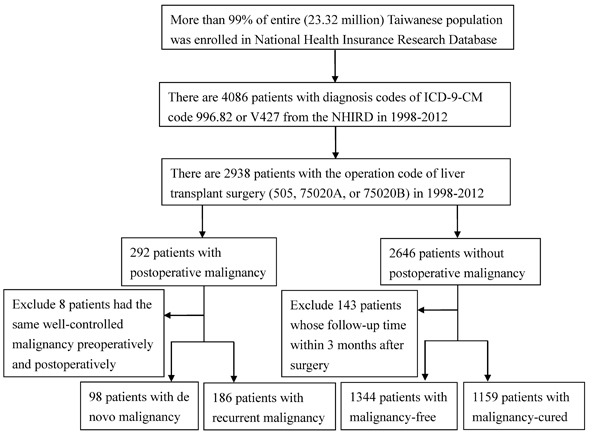
Flowchart ICD-9-CM, International Classification of Disease, Ninth Revision, Clinical Modification; NHIRD, National Health Insurance Research Database.

Of the 2938 LTRs, 2795 were followed for more than 3 months postoperatively. 284 LTRs had post-transplant malignancies, including *de novo* or recurrent malignancies, while 2503 LTRs followed for more than 3 months postoperatively, were malignancy free throughout the study period. Eight other patients had the same well-controlled malignancies pre- and postoperatively (Figure 1). The 284 LTRs that developed post-transplant malignancies, included 99 *de novo* cancers among 98 recipients and recurrent cancers in 186 recipients. The characteristics of all postoperative malignancies are summarized in Table 3. In *de novo* malignancies, the commonly affected organs or disease were the liver (19.39%), the oropharynx (19.39%; 6.12% of the oral cavity and 13.26% of the pharynx), non-Hodgkin's lymphoma (9.18%), the prostate (6.12%), and the esophagus (5.10%). Moreover, in recurrent malignancies, the commonly invaded organs were the liver (83.33%), lung (4.84%), bone and bone marrow (4.30%), and intrahepatic bile ducts (2.69%).

**Table 3:** Type, incidence, and malignancy-free interval after liver transplantation

	Number	Malignancy-free interval (years)		
		Median	Minimal	Maximal
***De novo* malignancy (*N*= 98)**				
Oral cavity	6	2.21	0.66	6.41
Pharynx	13	2.17	0.47	7.92
Esophagus	5	4.27	1.12	5.71
Stomach	3	6.59	2.25	6.66
Small intestine	1	2.11	2.11	2.11
Colon	2	1.54	1.26	1.83
Rectum	2	2.21	1.47	2.95
Liver	19	1.49	0.55	5.45
Ampulla of Vater	1	0.89	0.89	0.89
Gallbladder and extrahepatic bile ducts	1	4.96	4.96	4.96
Pancreas	1	3.95	3.95	3.95
Ill-defined sites within the digestive organs and peritoneum	1	14.08	14.08	14.08
Larynx	2	4.95	2.39	7.52
Lung	4	1.13	0.56	8.99
Pleura	1	1.89	1.89	1.89
Connective tissue	1	1.05	1.05	1.05
Skin	1	7.17	7.17	7.17
Breast	4	1.55	0.56	2.53
Kaposi's sarcoma	2	0.89	0.66	1.12
Cervix uteri	2	2.74	1.39	4.08
Prostate	6	3.09	0.85	9.47
Bladder	2	4.78	3.04	6.52
Kidney	1	0.57	0.57	0.57
Brain	3	1.27	0.99	4.89
Thyroid gland	1	7.44	7.44	7.44
Non-Hodgkin's lymphoma	9	2.27	0.58	4.90
Hodgkin's disease	1	3.53	3.53	3.53
Lymphoid leukemia	2	2.74	2.04	3.45
Myeloid leukemia	2	1.36	0.79	1.93
Total	99	2.11	0.47	14.08
**Recurrent malignancy (*N*= 186)**				
Liver	155	1.29	0.56	9.58
Intrahepatic bile ducts	5	2.15	1.65	2.71
lymph nodes	3	1.10	0.39	1.64
Lung	9	0.95	0.62	8.33
Pleura	1	2.47	2.47	2.47
Peritoneum	1	0.85	0.85	0.85
Other digestive organs and spleen	1	1.08	1.08	1.08
Brain and spinal cord	2	1.11	0.98	1.24
Bone and bone marrow	8	1.07	0.55	3.34
Other specified sites	1	1.99	1.99	1.99
Total	186	1.28	0.39	9.58

**Figure 2:**
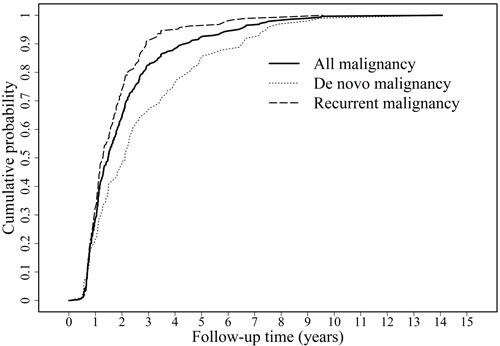
Cumulative cancer probability of de novo, recurrent, and all malignancies in the liver transplant recipients

The postoperative cumulative cancer probability examined using the Kaplan-Meier curve is presented in Figure 2. The cumulative cancer probability for LTRs at 1, 3, 5, and 10 postoperative years was 28.87% (82/284), 82.39% (234/284), 92.25% (262/284), and 99.65% (283/284), respectively. Furthermore, in *de novo* malignancies, the cumulative cancer probability at 1, 3, 5, and 10 postoperative years was 21.43% (21/98), 66.33% (65/98), 84.69% (83/98), and 98.98% (97/98), respectively. The mean time for the first diagnosis of postoperative cancer was 2.10 years (range, 0.39-14.28 years; median, 1.50). Recurrent malignancies developed faster than *de novo* malignancies. The cumulative cancer probability reached 90% at 2.91 years and 6.59 years in the recurrent and *de novo* malignancy groups, respectively.

In this study, 88 patients with postoperative malignancies died, and their mean survival time from diagnosis was 6.07 ± 0.17 (mean ± SE) years. For all patients in this cohort there were 428 deaths, and the mean survival time from diagnosis was 9.78 ± 0.09 (mean ± SE) years. The mortality rates were significantly higher in the LTRs than in the general population in Taiwan (http://www.ris.gov.tw/zh_TW/346).

### Risk factors associated with post-transplant malignancies

To analyze and compare the effects of specific risk factors, the LTRs were divided into two groups based on whether they had post-transplant malignancies in the cohort interval. Risk factors for each group are summarized in Table 4. Older LTRs were more predisposed to post-transplant malignancies than younger ones (hazard ratio, 1.025; 95% CI, 1.016 to 1.033; *p* < 0.0001). The risk of post-transplant malignancies was significantly higher in males than in females (hazard ratio, 1.51; 95% CI, 1.15 to 2.00; *p* = 0.0033). Patients with a past history of liver cirrhosis (hazard ratio, 1.46; 95% CI, 1.01 to 2.13; *p* = 0.0468) or chronic hepatitis (hazard ratio, 2.00; 95% CI, 1.06 to 3.77; *p* < 0.0329) were predisposed to malignancies. LTRs with hepatitis B, but not hepatitis C or alcoholic hepatitis, had a significantly elevated risk of cancer (*p* = 0.0085, 0.062, and 0.6235, respectively). Furthermore, recipients with biliary atresia (hazard ratio, 0.28; 95% CI, 0.16 to 0.50; *p* < 0.0001) or urea cycle metabolism disorders (*p* < 0.0001) appeared to be protected from malignancies. Peptic ulcers (hazard ratio, 1.53; 95% CI, 1.21 to 1.94; *p* = 0.0004) and diabetes mellitus (hazard ratio, 1.36; 95% CI, 1.04 to 1.79; *p* = 0.0264) were significantly more common in the malignancy group than in the non-malignancy group. In our study, the risk of postoperative malignancies was significantly higher in the LTRs with preoperative malignancies (hazard ratio, 3.82; 95% CI, 2.94 to 4.96; *p* < 0.0001). However, socioeconomic deprivation and other systemic diseases, such as hypertension, lung disease, or renal failure, were not associated with post-transplant malignancies. Postoperative sepsis, postoperative bleeding, epidural anesthesia, and morphine usage were not associated with postoperative malignancies.

**Table 4:** Analysis of risk factors for post-transplant malignancy

	Without malignancy after transplantation (*N* = 2503)		With malignancy after transplantation (*N* = 284)		Hazard Ratio	(95% CI)		*p*-value
	N or Mean	% or SD	N or Mean	% or SD				
Age^†^	45.90	±18.10	50.29	±13.36	1.03	(1.02,1.03)		<.0001
Gender								
Male	1742	69.60	219	77.11	1.51	(1.15,2.00)		0.0033
Female	761	30.40	65	22.89	-	-		-
Socioeconomic deprivation								
1(most deprived)	886	35.40	87	30.63	0.70	(0.35,1.40)		0.3113
2 (0-2.5k)	965	38.55	108	38.03	0.90	(0.45,1.79)		0.7628
3 (2.5k-5k)	481	19.22	64	22.54	1.07	(0.53,2.18)		0.8447
4 (5k-7.5k)	105	4.19	16	5.63	1.14	(0.50,2.59)		0.7608
5 (>7.5k)	66	2.64	9	3.17	-	-		-
Pre-operative cancer	1159	46.30	211	74.30	3.82	(2.94,4.96)		<.0001
Liver cirrhosis	2105	84.10	253	89.08	1.46	(1.01,2.13)		0.0468
Ascites	1196	47.78	134	47.18	0.85	(0.67,1.07)		0.1684
Chronic hepatitis	2342	93.57	274	96.48	2.00	(1.06,3.77)		0.0329
Hepatitis B	1196	47.78	156	54.93	1.37	(1.08,1.73)		0.0085
Hepatitis C	557	22.25	69	24.30	1.30	(0.99,1.70)		0.062
Alcoholic hepatitis	482	19.26	51	17.96	1.08	(0.80,1.46)		0.6235
Hepatic coma	849	33.92	93	32.75	0.92	(0.71,1.17)		0.4799
Hypertension	505	20.18	53	18.66	1.14	(0.85,1.54)		0.3852
Diabetes mellitus	512	20.46	67	23.59	1.36	(1.04,1.79)		0.0264
Peptic ulcer	1193	47.66	161	56.69	1.53	(1.21,1.94)		0.0004
Renal failure	87	3.48	13	4.58	1.35	(0.78,1.35)		0.2885
Pulmonary disease	354	14.14	44	15.49	1.23	(0.89,1.70)		0.2029
Hypercholesterolemia	331	13.22	26	9.15	0.80	(0.53,1.20)		0.2731
Biliary atresia	272	10.87	12	4.23	0.28	(0.16,0.50)		<.0001
Wilson's disease	34	1.36	1	0.35	0.20	(0.20,1.35)		0.0979
Glycogen storage disease	26	1.04	1	0.35	0.25	(0.03,1.84)		0.1734
Urea cycle metabolism disorders	16	0.64	0	0.00	0.00	(0.00,0.00)		<.0001
Obese	10	0.40	2	0.70	1.91	(0.50,7.39)		0.3466
Bacteremia	137	5.47	12	4.23	0.60	(0.34,1.09)		0.0926
Postoperative bleeding	126	5.03	21	7.39	1.16	(0.74,1.81)		0.5245
Epidural anesthesia	11	0.44	1	0.35	0.62	(0.09,4.39)		0.6290
Morphine	1246	49.78	137	48.24	0.81	(0.65,1.03)		0.0797

† Values are mean and standard deviation.

Hazard ratios are for malignancy as compared with non-malignancy after transplantation. CI denotes confidence interval.

### Recurrent malignancies considerably increased mortality in the LTRs

**Figure 3:**
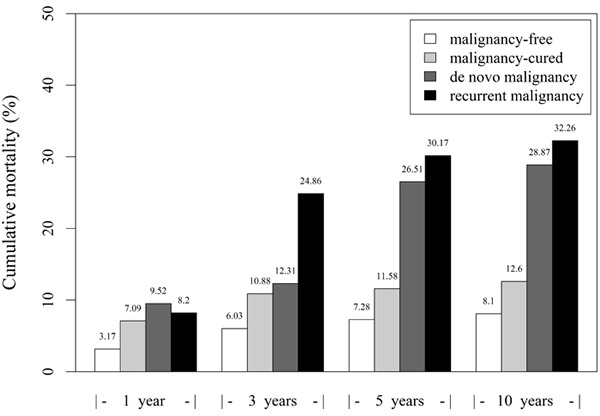
Effects of post-transplant malignancies on the liver transplant recipients by bar plot

The results revealed increased mortality in the LTRs with postoperative malignancies, and the LTRs with recurrent malignancies had higher mortality than did those with no postoperative malignancies (RR = 3.15, CI = 2.49 to 4.00) or with *de novo* malignancies (RR = 1.13, CI = 0.78 to 1.64), respectively. The result suggested that compared with the de novo or postoperative cancer-free groups, the recurrent group considerably increased LTR mortality (Figure 3).

Overall mortality among the four groups were 5.13% (143/2787), 9.36% (261/2787), and 11.12% (310/2787) at 1, 3, and 5 years, respectively. The mortality rates of the *de novo* group were 9.52% (2/21), 12.31% (8/65), and 26.51% (22/83) at 1, 3, and 5 years, respectively. Furthermore, in the recurrent group, the mortality rates were 8.20% (5/61), 24.85% (42/169), and 30.17% (54/179) at 1, 3, and 5 years, respectively. Moreover, the 10-year patient mortality rates in the malignancy-free, malignancy-cured, *de novo*, and recurrent groups were 8.10% (109/1345), 12.60% (146/1159), 28.87% (28/97) and 32.26% (60/186), respectively. These results indicated a strong association of mortality with postoperative malignancies; the LTRs with postoperative malignancies revealed higher mortality, particularly considering those with recurrent malignancies.

## DISCUSSION

In this retrospective study of 2938 LTRs, *de novo* malignancies occurred in 3.34% of recipients at a mean relapse time of 5.71 years postoperatively. The recurrence rate among the patients with pre-existing malignancies was 12.92% (186/1440) at a mean elapsed time of 1.71 years. Moreover, the SIR of postoperative *de novo* cancers was 2.17-fold (95% CI, 1.76 to 2.64) compared to the general Taiwanese population. Our data are consistent with those of previous studies that reported the tendency to develop malignancies after solid organ transplantation, in which the relative risk ratio was estimated to be between 2.1 and 4.3 [5, 7–9].

Our present study revealed that 65.49% (186/284) of post-transplant malignancies were recurrent. The most common relapse site was the liver, and distal metastasis occurred at the lung and bone or bone marrow. The most common *de novo* malignancies in the LTRs were HCC (SIR: 3.15, CI: 1.90-4.92), oropharyngeal cancer (SIR: 4.61, CI: 2.77-7.19), and non-Hodgkin's lymphoma (SIR: 8.42, CI: 3.84-15.98), which were all confirmed as infection-related malignancies. The results differed from the prevalence of cancer types in Taiwanese population, in whom the most common malignancies were observed at the colon or rectum, liver, lung, and breast, in that order. Iatrogenic immunosuppression is associated with oncogenesis and immune deficient patients with AIDS also showed significantly higher SIRs for infection-related malignancies than the general population. HCC is associated with hepatitis B and C virus infection; oropharyngeal cancer is HPV related; and non-Hodgkin lymphoma is Epstein-Barr virus related [10]. In addition, esophageal and gastric cancers were *Helicobacter pylori* related [10, 17], and comprised 8.25% of *de novo* malignancies observed in LTRs. Multivariate analysis also revealed that post-transplant malignancies were significantly associated with hepatitis B, but not with hepatitis C or alcoholic hepatitis. Preoperative hepatitis virus B-infected patients appeared predisposed to oncogenesis after immunosuppression. Furthermore, *H. pylori* infection is a major risk factor for peptic ulcers, infecting 90%-100% and 60%-90% of patients with gastric and duodenal ulcers, respectively [18, 19]. We could not directly evaluate *H. pylori* infection in the LTRs; however, we observed an association between patients with peptic ulcers and malignancies. Vigorously treating peptic ulcers before administering immunosuppressant might reduce post-transplant malignancies, particularly esophageal and gastric cancers; however, further studies are required to support this hypothesis. In conclusion, our results reveal the effect of immunosuppressive treatment on postoperative malignancies in LTRs. A similar risk of cancer in the LTRs and patients with AIDS suggests that immune deficiency is, at least partially, responsible for the increased risk.

In this study of the Chinese ethnicity, skin cancer was relatively rare, estimated to be 1.01% in the *de novo* group; however, it is relatively common in *de novo* malignancies reported in western countries [20]. Skin cancer accounted for 38.67% of postoperative malignancies in a similar study of the Swedish population [20]. Among post-transplant *de novo* malignancies, 5.3% of American recipients and 4.21% of Australian recipients reported malignancies among solid organs, including the liver, kidneys, heart, and lungs [8, 21]. Furthermore, the postoperative melanoma incidence has been reported as 2%-4% in Swedish and Italian populations [9, 20]; however, no melanoma developed in our cohort.

Age and sex were the major risk factors for post-transplant malignancies, thus warranting detailed screening for malignancies. Patients with comorbidities of preoperative liver cirrhosis, diabetes mellitus, and pre-existing cancers were also high-risk populations. Advanced liver fibrosis is a confirmed risk factor for HCC carcinogenesis [22]. Pre-existing malignancies are recurrent in nature, and this factor may be apparent under the effect of tumor surveillance escape as natural killer cells are inhibited by immunosuppressant. Patients with biliary atresia or urea cycle metabolism disorders appear to have a decreased risk of post-transplant cancers. These findings should not be inclusion or exclusion criteria for liver transplant candidates; instead, they must remind clinicians to reduce the immunosuppressant dose and to carefully screen for risk.

A low socioeconomic status is correlated with the incidence and mortality of various malignancies, such as HCC, esophageal, breast, and colorectal cancer [23–28]. Our present data revealed no correlation between socioeconomic status and incidence of post-transplant malignancies (*p* = 0.3312). This may have contributed to earlier diagnosis and optimal treatment without the barriers of socioeconomic status because of the patient care provided by the NHI program. This observation was also supported by the relatively lower SIR (2.17-fold) of de novo cancer in our cohort compared with that reported by previous studies (2.1 to 4.3-fold). Iwasaki et al. reported the possible influence of volatile anesthetics on cancer cell biology and metastatic potential *in vitro* study [29]. However, NHIRD cannot provide any record of anesthetic used in surgery.

The recipients with post-transplant malignancies had a significantly higher mortality; those with recurrent malignancies survived less frequently than those with de novo malignancies. Our data revealed that the mortality of malignancy-cured patients was slightly inferior to that of malignancy-free patients, but lower than that of the *de novo* and recurrent groups. The 10-year patient mortality rates were 8.1% and 12.6% in the malignancy-free and malignancy-cured groups, supporting the assumption that liver transplantation is a favorable treatment option for localized HCC with no recurrence. Moreover, careful patient selection and an immunosuppression-minimizing strategy are warranted.

The study has some limitations. First, the NHIRD could not provide data on risk factors, such as lifestyle, living or working environment, and family history [30]. Smoking, alcohol consumption, environmental toxins or pollution, and genetic inheritance play crucial roles in oncogenesis. The effects of latency and dosage on these risk factors were also unknown. In addition, the average follow-up interval was relatively short (3.79 ± 3.26 years) and may cause the underestimation of late-onset carcinogenesis. Second, although the present study revealed risk factors for post-transplant malignancies, the actual causality between them remains unconfirmed. Third, in addition to immunosuppressant-induced malignancy, circulating cancer cells before transplantation, surgical manipulation-induced tumor seeding, and tumorigenesis tendency of recipients were possible risk factors for recurrence of post-transplant HCC unaccounted for in our data.
